# Association Between Passive Smoking and Menstrual Discomfort: A Cross-Sectional Study of 2,571 Non-smoking Chinese Nurses

**DOI:** 10.3389/fpubh.2022.889254

**Published:** 2022-05-26

**Authors:** Shi-qi Xiao, Lin-lin Xing, Qi-jun Wu, Tian-hui Xia, Tong-tong Fu, Ying Guo, Xin-ying Yu, Xiang-hong Sun, Hui-ling Feng, Li Gai, Yi-wei Xu, Chun-ling Xia, Ling Fan

**Affiliations:** ^1^Department of Nursing, Shengjing Hospital of China Medical University, Shenyang, China; ^2^Department of Clinical Epidemiology, Shengjing Hospital of China Medical University, Shenyang, China; ^3^Department of Nursing, China Medical University, Shenyang, China; ^4^Department of Nursing, Hebei University of Chinese Medicine, Shijiazhuang, China

**Keywords:** dysmenorrhea, menstrual discomfort, nurses' health, passive smoking, cross-sectional study

## Abstract

**Introduction:**

Menstrual discomfort affects women's quality of life, which is an important public health issue. Evidence confirming the link between passive smoking and menstrual discomfort is limited. Therefore, the aim of this study is to investigate the aforementioned topic on the basis of a cross-sectional study of 2,571 non-smoking Chinese nurses.

**Methods:**

Demographic information and passive smoking were assessed using a self-administered questionnaire. Menstrual discomfort was characterized as dysmenorrhea, illness or weakness, bed rest, and restlessness during menstruation, which was assessed using a modified version of the Cornell Medical Index-Health Questionnaire. Multivariate-adjusted odds ratio (OR) and 95% confidence intervals (CIs) were estimated using the logistic regression model.

**Results:**

A total of 1:195 nurses (46.48%) were exposed to passive smoking. Compared with non-passive smoking nurses, passive smoking nurses were more likely to have menstrual discomfort symptoms (72.38 vs. 64.39%), especially symptoms of dysmenorrhea (49.54 vs. 42.08%), illnesses or weakness (48.28 vs. 42.08%), and restlessness during menstruation (53.05 vs. 46.22%). Exposure to passive smoking was significantly associated with menstrual discomfort (OR = 1.41, 95%CI: 1.19–1.67), especially symptoms of dysmenorrhea (OR = 1.32, 95%CI: 1.13–1.56), illness or weakness (OR = 1.24, 95%CI: 1.06–1.46), and restlessness (OR = 1.26, 95%CI: 1.08–1.48) during menstruation. The subgroup analyses, stratified by age, children, and marital status, agreed with the main findings.

**Conclusions:**

Exposure to passive smoking was related to symptoms of dysmenorrhea and menstrual discomfort.

## Introduction

Dysmenorrhea is a severe lower abdominal pain that occurs in women during menstruation, which usually has the nature of cramps and may be radiated to the thigh or lower spine, often accompanied by vomiting, headache, back pain, diarrhea, fatigue, and even syncope symptoms (1). Moreover, getting sick or feeling weak during menstruation, being bedridden during menstrual periods, and feeling restless during menstrual periods are also considered symptoms of menstrual discomfort. These conditions affect women's daily life, work, and study to varying degrees and reduce their quality of life (2, 3). According to statistics, the prevalence of primary dysmenorrhea in women of childbearing age is about 45–95%, of which 2–29% are severe dysmenorrhea, which has become an important public health problem facing women's health (4, 5).

Although previous evidence has suggested a high incidence of dysmenorrhea, the factors that influence dysmenorrhea in reproductive longevity are not fully understood. Recent epidemiological studies have identified several risk factors for dysmenorrhea, including family history of dysmenorrhea, age at menarche, oral contraceptive use, dietary habits, caffeine intake, smoking, and stress (4, 6–10). Smoking is one of the modifiable risk factors for dysmenorrhea. Moreover, due to the lower smoking rates among women and children, studies on their exposure to second-hand smoke (SHS) are increasing, suggesting that passive smoking is a more severe public health problem than active smoking in these groups (11).

Passive smoking, also known as SHS, is one of the most serious public health threats. Data show that ~65.1% of non-smokers in 12 provinces in China are exposed to SHS, whereas 44.9% of adults (515.0 million adults) are exposed to SHS at home (12). Moreover, many studies have indicated that passive smoking is closely associated with cardiovascular disease (13, 14), allergy and asthma-like symptoms (3), breast cancer, reproductive system disease (15, 16), and high blood pressure (17, 18). However, only a few studies have examined the relationship between SHS exposure and dysmenorrhea (19, 20).

Given the limited and controversial evidence about the relationship between passive smoking and dysmenorrhea, we performed a cross-sectional study of 2,571 non-smoking Chinese nurses, to further elucidate the aforementioned topic.

## Methods

### Study Participants

The participants included in this study were recruited from the Health Evaluation of Occupational Nurses (21). It is designed to be a cohort study with long-term follow-up in 2019 to evaluate the effects of environmental and genetic interactions on Chinese nurses' health, held at the Shengjing Hospital of China Medical University in Shenyang, Liaoning Province. All of the registered nurses in service were enrolled in the cohort study, except those who were absent from the annual health examination due to a sick leave or personal leave. They filled out a self-administered questionnaire about their health status and lifestyle every year and donated a peripheral blood sample during the annual health examination every 2 years. All of the nurses participated in the study voluntarily and provided their written informed consent. The Health Evaluation of Occupational Nurses study was approved by the Ethics Committee of Shengjing Hospital of China Medical University (2018PS09K).

By 2021, a total of 3,469 nurses had participated in this cohort study. Recently, we used the baseline data (2019) to conduct the present study. We first excluded male nurses (*n* = 131), to limit the participants only to female nurses. Subsequently, we excluded those who had the following characteristics: perimenopausal and post-menopausal (*n* = 161), age more than 45 years (*n* = 55), and presence of uterine fibroids (*n* = 325), since they were no longer menstruating or their menstrual symptoms might be affected by the aforementioned factors. We further excluded participants for active smoking (*n* = 36) and those exposure to passive smoking were unavailable (*n* = 190). Finally, a total of 2,571 participants were eligible for analysis.

### Exposure Assessment

We used a self-administered questionnaire that included five parts: the sociodemographic questionnaire, nurses' stressful work sources, healthy life promotion methods, nurses' job satisfaction, and the Cornell Medical Index (CMI)-Health Questionnaire. The CMI questionnaire is a self-administered questionnaire to provide a standardized health check, which has been used widely as a sensitive indicator of health status. The CMI used in this study was a modified Chinese version consisting of 195 questions, each question had two answers, “yes” or “no,” corresponding to a “0” or “1” score. The score range by the CMI was 0–195, with higher scores indicating poorer levels of health status. The CMI questionnaire in this study offered excellent reliability (Chronbach's α = 0.970). The detail descriptions of these characteristics were introduced in the article by Feng HL (22).

### Active and Passive Smoking

People who had smoked at least one cigarette per day for over 6 months in their lifetime were defined as active smokers (20). Exposure to passive smoking was evaluated by a single question with two response options [“yes” and “no” (23)]: “Have you inhaled the smoke of cigarettes from others (i.e., smokers) at home, in an office, or elsewhere for more than 15 min 1 day a week on average in the last year?”

### Menstrual Discomfort

Menstrual discomfort symptoms were assessed by a revised edition of the CMI-Health Questionnaire that comprised 195 questions (24). The questionnaire was classified into two parts: the A–L collection was related to physical morbidity, and the M–R collection referred to psychological disorders. The following questions describe the women's menstrual discomfort symptoms with two response options (“yes” and “no” each):

- “Do you often have dysmenorrhea (abdominal pain before, during, and after menstruation)?” (97th question)
- “Do you often get sick or feel weak during your menstrual period?” (98th question)
- “Do you often lie in bed during your menstrual period, or do you have vaginal bleeding outside your menstrual period?” (99th question)
- “Do you often feel restless during your menstrual period?” (101st question)

If the answer to any of these questions is yes, we will define it as menstrual discomfort because menstrual discomfort currently has no clear definition. Menstrual discomfort was also referred to as dysmenorrhea, illness or weakness, bed rest, and restlessness.

### Statistical Analysis

The participants' characteristics were evaluated using descriptive statistics. We used the mean ± standard deviation to report the continuous variables as well as counts and percentages to represent the categorical variables. Independent sample Student's *t*-tests were used to compare the differences in the continuous variables between the groups, whereas the chi-square test was used to determine the relationship between the categorical variables. To evaluate the association between passive smoking and menstrual discomfort symptoms, we conducted logistic regression analyses to calculate the risk estimates. First, we analyzed the association between passive smoking and menstrual discomfort symptoms as a whole and then analyzed the association between passive smoking and each symptom in detail. In Model 1, we only adjusted for age. In Model 2, we further adjusted for age (≤ 30 or >30 years old), body mass index (BMI) (normal, underweight, and overweight), department (Medical, Surgical, Obstetrics and Gynecology, Pediatrics, Outpatient and emergency, and ICU/Operating Room), marital status (married or other), professional title (nurse, senior nurse, supervisor, or above), shift work (yes or no), children (yes or no), income (≤9,000 or >9,000 Yuan), alcohol consumption (yes or no), tea consumption (yes or no), and coffee consumption (yes or no). We calculated the adjusted risk estimates in the subgroup analyses and conducted interaction analyses stratified by age, children, and marital status additionally. All the analyses were conducted using SPSS 21.0 (IBM, Armonk, New York, USA). All statistical tests were two-sided, and a *p-*value of <0.05 was considered statistically significant.

## Results

### Basic Characteristics of the Participants

The basic characteristics of the participants stratified by passive smoking are shown in Table 1. A total of 1,195 (46.48%) nurses were exposed to passive smoking. Nurses with passive smoking exposure were more likely to be surgical nurses and had higher alcohol, tea, and coffee consumption than non-passive smokers (*P* < 0.05).

**Table 1:** Basic characteristics of the participants according to passive smoking exposure (*n* = 2,571).

	**Total**	**Non-SHS**	**SHS**	***P*-value**
	***n* = 2,571**,	***n* = 1,376**,	***n* = 1,195**,	
	**mean ±SD/**	**mean ±SD/**	**mean ±SD/**	
	***n* (%)**	***n* (%)**	***n* (%)**	
Age (year)	31.57 ± 4.28	31.65 ± 4.34	31.48 ± 4.22	0.33
≤ 30	1,022 (39.75)	535 (38.88)	487 (40.75)	
>30	1,549 (60.25)	841 (61.12)	708 (59.25)	
BMI (kg/m^2^)	21.66 ±2.89	21.59 ±2.85	21.73 ±2.93	0.55
Normal	1,831 (71.22)	987 (71.73)	844 (70.63)	
Underweight	276 (10.74)	151 (10.97)	125 (10.46)	
Overweight	464 (18.05)	238 (17.30)	226 (18.91)	
Department				<0.05
Medical	675 (26.25)	358 (26.02)	317 (26.53)	
Surgical	511 (19.88)	252 (18.31)	259 (21.67)	
Obstetrics and gynecology	324 (12.60)	171 (12.43)	153 (12.80)	
Pediatrics	297 (11.55)	186 (13.52)	111 (9.29)	
Outpatient and emergency	415 (16.14)	235 (17.08)	180 (15.06)	
ICU/Operating Room	349 (13.57)	174 (12.65)	175 (14.64)	
Education				0.58
Below bachelor	228 (8.87)	118 (8.58)	110 (9.21)	
Bachelor or above	2,343 (91.13)	1,258 (91.42)	1,085 (90.79)	
Marital status				0.96
Married	1,918 (74.60)	1,026 (74.56)	892 (74.64)	
Others	653 (25.40)	350 (25.44)	303 (25.36)	
Professional title				0.23
Nurse	311 (12.10)	159 (11.56)	152 (12.72)	
Senior nurse	2,093 (81.41)	1,118 (81.25)	975 (81.59)	
Supervisor or above	167 (6.50)	99 (7.19)	68 (5.69)	
Shift work				0.30
No	911 (35.43)	500 (36.34)	411 (34.39)	
Yes	1,660 (64.57)	876 (63.66)	784 (65.61)	
Children				0.56
No	957 (37.22)	505 (36.70)	452 (37.82)	
Yes	1,614 (62.78)	871 (63.30)	743 (62.18)	
Income (yuan)				0.71
≤ 9,000	1,690 (65.73)	900 (65.41)	790 (66.11)	
>9,000	881 (34.27)	476 (34.59)	405 (33.89)	
Exercise three times a week				0.24
No	1,356 (52.74)	711 (51.67)	645 (53.97)	
Yes	1,215 (47.26)	665 (48.33)	550 (46.03)	
Alcohol consumption				<0.05
No	2,224 (86.50)	1,235 (89.75)	989 (82.76)	
Yes	347 (13.50)	141 (10.25)	206 (17.24)	
Tea consumption				<0.05
No	2,093 (81.41)	1,150 (83.58)	943 (78.91)	
Yes	478 (18.59)	226 (16.42)	252 (21.09)	
Coffee consumption				<0.05
No	2,047 (79.62)	1,122 (81.54)	925 (77.41)	
Yes	524 (20.38)	254 (18.46)	270 (22.59)	

*SHS, second-hand smoke; SD, standard deviation*.

### Association Between Passive Smoking and Menstrual Discomfort Symptoms

Compared with non-passive smokers, nurses with passive smoking exposure were more prone to have menstrual discomfort, especially symptoms of dysmenorrhea, illness or weakness, and restlessness during menstruation (Table 2). We found that nurses exposed to passive smoking were more likely to have menstrual discomfort in general than non-passive smokers (OR = 1.41, 95%CI: 1.19–1.67) (Table 3). Individual analysis of the symptoms menstrual discomfort revealed that nurses with passive smoking exposure were more likely to have symptoms of dysmenorrhea (OR = 1.32, 95%CI: 1.13–1.56), illness or weakness (OR = 1.24, 95%CI: 1.06–1.46), and restlessness (OR = 1.26, 95%CI: 1.08–1.48) during menstruation than non-passive smokers. Furthermore, the results of the subgroup analyses were mainly consistent with the main findings. No significant interactions were found in the subgroup analyses (Table 4).

**Table 2:** Association between SHS and menstrual discomfort symptoms (*n* = 2,571).

	**Non-SHS**	**SHS**	***P*-value**
	***n* = 1,376**,	***n* = 1,195**,	
	***n* (%)**	***n* (%)**	
Menstrual discomfort			<0.05
No	490 (35.61)	330 (27.62)	
Yes	886 (64.39)	865 (72.38)	
Dysmenorrhea			<0.05
No	797 (57.92)	603 (50.46)	
Yes	579 (42.08)	592 (49.54)	
Illness or weakness			<0.05
No	797 (57.92)	618 (51.72)	
Yes	579 (42.08)	577 (48.28)	
Bed rest			0.30
No	1,276 (92.73)	1,095 (91.63)	
Yes	100 (7.27)	100 (8.37)	
Restlessness			<0.05
No	740 (53.78)	561 (46.95)	
Yes	636 (46.22)	634 (53.05)	

*SHS, second-hand smoke; OR, odds ratio; CI, confidence interval; Adjusted^1^, adjusted for age only; Adjusted^2^, adjusted for age, BMI, department, marital status, professional title, shift work, children, income, alcohol consumption, tea consumption, and coffee consumption*.

*ORs and 95% CIs for nurses' menstrual discomfort symptoms according to exposure to SHS among nonsmokers (n = 2,571)*.

**Table 3:** ORs and 95% CIs for nurses' menstrual discomfort symptoms according to exposure to SHS among nonsmokers (*n* = 2,571).

	**Non-SHS**,	**SHS**,
	***n* = 1,376**	***n* = 1,195**
Menstrual discomfort		
Crude OR (95% CI)	1.00 (Ref)	1.45 (1.26–1.72)
Age-adjusted OR (95% CI)	1.00 (Ref)	1.44 (1.22–1.71)
Multivariate-adjusted OR^*^ (95% CI)	1.00 (Ref)	1.41 (1.19–1.67)
Dysmenorrhea		
Crude OR (95% CI)	1.00 (Ref)	1.35 (1.16–1.58)
Age-adjusted OR (95% CI)	1.00 (Ref)	1.35 (1.15–1.57)
Multivariate-adjusted OR^*^ (95% CI)	1.00 (Ref)	1.32 (1.13–1.56)
Illness or weakness		
Crude OR (95% CI)	1.00 (Ref)	1.29 (1.10–1.50)
Age-adjusted OR (95% CI)	1.00 (Ref)	1.29 (1.10–1.50)
Multivariate-adjusted OR^*^ (95% CI)	1.00 (Ref)	1.24 (1.06–1.46)
Bed rest		
Crude OR (95% CI)	1.00 (Ref)	1.17 (0.87–1.56)
Age-adjusted OR (95% CI)	1.00 (Ref)	1.17 (0.87–1.56)
Multivariate-adjusted OR^*^(95% CI)	1.00 (Ref)	1.10 (0.82–1.47)
Restlessness		
Crude OR (95% CI)	1.00 (Ref)	1.32 (1.13–1.54)
Age-adjusted OR (95% CI)	1.00 (Ref)	1.31 (1.12–1.53)
Multivariate-adjusted OR^*^ (95% CI)	1.00 (Ref)	1.26 (1.08–1.48)

SHS, second-hand smoke; OR, odds ratio; CI, confidence interval; Multivariate-adjusted OR^*^, adjusted for age, BMI, department, marital status, professional title, shift work, children, income, alcohol consumption, tea consumption, and coffee consumption.

**Table 4:** Subgroup analysis and interaction analysis.

	**Menstrual discomfort**	*P*-value^*^	**Dysmenorrhea**	*P*-value^*^	**Illness or weakness**	*P*-value^*^	**Bed rest**	*P*-value^*^	**Restlessness**	*P*-value^*^
Age (year)		0.32		0.90		0.92		0.15		0.91
≤ 30	1.59 (1.20–2.12)		1.35 (1.05–1.74)		1.25 (0.97–1.61)		1.40 (0.86–2.26)		1.26 (0.98–1.62)	
>30	1.33 (1.07–1.65)		1.32 (1.07–1.63)		1.27 (1.03–1.56)		0.95 (0.65–1.39)		1.28 (1.05–1.58)	
Children		0.46		0.97		0.32		0.31		0.74
No	1.56 (1.14–2.11)		1.34 (1.03–1.75)		1.40 (1.08–1.82)		1.27 (0.79–2.03)		1.30 (0.99–1.69)	
Yes	1.36 (1.10–1.68)		1.33 (1.08–1.63)		1.18 (0.96–1.45)		0.99 (0.68–1.46)		1.25 (1.03–1.53)	
Marital status		0.57		0.24		0.70		0.22		0.27
Married	1.36 (1.13–1.67)		1.41 (1.17–1.70)		1.27 (1.05–1.52)		1.01 (0.72–1.42)		1.20 (0.99–1.44)	
Others	1.56 (1.08–2.26)		1.13 (0.82–1.57)		1.22 (0.89–1.68)		1.51 (0.81–2.85)		1.52 (1.10–2.10)	

P–value^*^ for interaction.

## Discussion

To our knowledge, this is the first large cross-sectional study to investigate the association between passive smoking and menstrual discomfort in Chinese nurses, which used the CMI-Health Questionnaire as a measurement indicator. We found that passive smoking was associated with menstrual discomfort, especially symptoms of dysmenorrhea, illness or weakness, and restlessness during menstruation. These findings still agree with those of the subgroup analyses.

Our findings are consistent with several previous studies showing that women with SHS exposure had significantly more severe menstrual symptoms than those without SHS exposure (19, 20, 25). For example, one study in China showed that the adjusted OR of dysmenorrhea associated with “low,” “middle,” and “high” SHS exposure vs. no exposure was 1.1 (95%CI: 0.5–2.6), 2.5 (95%CI: 0.9–6.7), and 3.1 (95%CI: 1.2–8.3), respectively (20). Furthermore, Nali et al. found that CYP1A1MspI and HincII genotypes modified the association between passive smoking and primary dysmenorrhea, based on 1,645 female textile workers (25). However, due to the small sample size, these studies did not conduct subgroup analysis to further understand this topic. Our study adds to previous findings by adjusting for different potential confounders and evaluating the symptoms related to menstruation using the CMI-Health Questionnaire. In addition, dysmenorrhea, menstrual illnesses, weakness, and restlessness were also investigated.

The exact biological mechanisms by which SHS may affect dysmenorrhea have not yet been fully investigated. Some researchers have suggested that nicotine, which is often detected in women with dysmenorrhea, is a vasoconstrictor that reduces blood flow to the lining of the uterus (26). Moreover, the CYP1A1 MspI variant GENE C/C6235 or the CYP1A1 HincII wild gene Ile/Ile462 was found in the DNA test of women with passive smoking exposure, which reduced their ability to convert toxic metabolites in cigarette smoke into less harmful hydrophilic compounds and significantly increased the risk of dysmenorrhea. However, these genes were less common in women who are non-passive smokers (25, 27, 28). Researchers have also suggested that nicotine may negatively affect the estrogen levels of women, which could lead to dysmenorrhea (29, 30).

Our study has several strengths. First, this is the first study to investigate the relationship between passive smoking and dysmenorrhea in nurses. Of note, their working environment is smoke-free, so this study can well-investigate the relationship between passive smoking in non-workplace environments and dysmenorrhea. Second, our study included a sample size of 2,571 to allow further analysis of numerous subgroups. Moreover, the results of these subgroup analyses agree with the main results, making our results more scientifically credible. Third, the nurses' health knowledge and ability can provide comprehensive and accurate information, which ensures scientific and accurate results to a certain extent. Finally, this study used the CMI-Health Questionnaire to measure the symptoms related to the menstruation of nurses, which is limited to not only dysmenorrhea but also the symptoms of menstrual discomfort and made a corresponding analysis and discussion.

However, this study has several limitations. First, this is a cross-sectional study and could not determine the cause and effect among some factors. Second, our measurement of potential confounders was based on a self-report questionnaire rather than biometric measures. However, nurses with a certain medical background were selected in this study to ensure the reliability of the results to a certain extent. Third, we did not collect the dose and duration of exposure to passive smoking in the baseline questionnaire, which restricts further investigation of the relationship between the dose and length of exposure to passive smoking and dysmenorrhea.

Dysmenorrhea has a significant negative impact on women and has become an important public health problem. Our study demonstrated a significant association between passive smoking and dysmenorrhea as well as menstrual discomfort symptoms.

## Data Availability Statement

The original contributions presented in the study are included in the article/Supplementary Material, further inquiries can be directed to the corresponding author/s.

## Ethics Statement

This project was funded by the Department of Science and Technology of Liaoning Province (Grant Number 2018225005). The patients/participants provided their written informed consent to participate in this study.

## Author Contributions

LF, S-qX, L-lX, and C-lX helped with the conceptualization of the study and funding acquisition. T-hX, H-lF, LG, and Q-jW contributed the materials and analysis tools. T-tF, X-yY, X-hS, Y-wX, and YG helped in the data collection and field operations. All authors helped to prepare the manuscript and approved the submitted version.

## Funding

This project was funded by the Department of Science and Technology of Liaoning Province (Grant Number 2018225005).

## Conflict of Interest

The authors declare that the research was conducted in the absence of any commercial or financial relationships that could be construed as a potential conflict of interest.

## Publisher's Note

All claims expressed in this article are solely those of the authors and do not necessarily represent those of their affiliated organizations, or those of the publisher, the editors and the reviewers. Any product that may be evaluated in this article, or claim that may be made by its manufacturer, is not guaranteed or endorsed by the publisher.
