# Supplementary figures and images for: Association Between Passive Smoking and Menstrual Discomfort: A Cross-Sectional Study of 2,571 Non-smoking Chinese Nurses
Source: Front Public Health. 2022 May 26;10:889254. doi: 10.3389/fpubh.2022.889254 (PMC9204475; doi:10.3389/fpubh.2022.889254)

### Flow chart of screening nurse participants

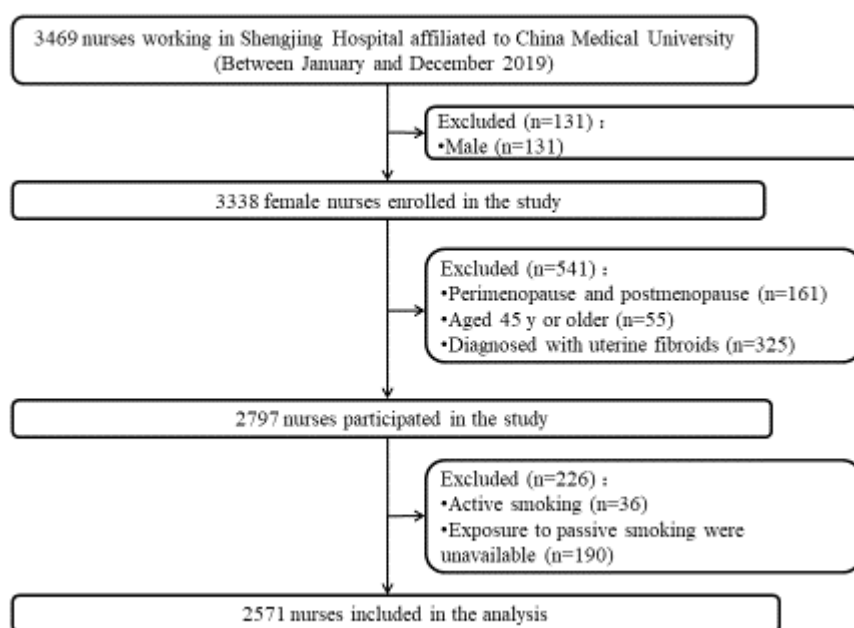

Supplement: Supplementary file 1 [file Image_1.pdf]
