# Supplementary material for: Association Between Passive Smoking and Menstrual Discomfort: A Cross-Sectional Study of 2,571 Non-smoking Chinese Nurses
Source: Front Public Health. 2022 May 26;10:889254. doi: 10.3389/fpubh.2022.889254 (PMC9204475; doi:10.3389/fpubh.2022.889254)
Supplement: Supplementary file 2 [file Presentation_1.pdf]

Cornell Medical Index Questionnaire (CMI)

A

1. Do you need to wear glasses when reading the newspaper?
2. Do you need to wear glasses to see far away?
3. Do you often have the phenomenon of temporary blackening (decreased vision or invisible things)?
4. Do you have frequent blinks and tears?
5. Do your eyes often hurt or do you often have blurry vision?
6. Are your eyes often red or inflamed?
7. Are you deaf (poor hearing)?
8. Have you ever had otitis media or ear discharge?
9. Do you often have tinnitus? (There are various sounds in your ears that affect your hearing)

B

10. Do you often have to cough to clear your throat?
11. Do you often experience a stuffy throat (feel like something is in your throat)?
12. Do you often sneeze?
13. Do you feel your nose is always blocked?
14. Do you often have a runny nose?
15. Do you sometimes have bad nosebleeds?
16. Do you often have bad colds or do you often have a sore throat and swollen tonsils?
17. Do you often have severe chronic bronchitis (coughing during a cold, spitting for a long time)?
18. Do you have to stay in bed when you have a cold or do you often spit?
19. Does frequent cold make you sick all winter?
20. Do you have allergic asthma (asthma that is triggered by certain allergic factors, such as pollen)?
21. Do you have asthma (recurrent, transient dyspnea with wheezing)?
22. Are you often troubled by coughing?
23. Have you ever coughed up blood?
24. Do you have heavy night sweats (sweating while sleeping and ending when you wake up)?
25. Have you ever suffered from chronic respiratory diseases (chronic bronchitis, bronchiectasis, emphysema) or have you recently had a low-grade fever (fever) (37-38 degrees)?
26. Have you ever had a malignant infectious disease (hepatitis B, etc.)?
27. Have you ever lived with someone who has a serious infectious

disease?

C

28. Did the doctor say you have high blood pressure?
29. Did the doctor say your blood pressure is very low?
30. Do you have chest or heart pain?
31. Do you often experience tachycardia (tachycardia)?
32. Do you often experience palpitations (a feeling of your heart beating when you are calm) or do you often feel your pulse stops beating?
33. Do you often have trouble breathing?
34. Are you more prone to shortness of breath (breathing) than others?
35. Do you sometimes feel short of breath even when sitting?
36. Do you often have severe lower extremity edema?
37. Are you troubled by cold hands and feet even on hot days?
38. Do you often have leg cramps?
39. Did the doctor say you have something wrong with your heart?
40. Are there any heart patients in your family?

D

41. Have you lost more than half of your teeth?
42. Are you troubled by bleeding gums (gum)?
43. Do you often have severe toothaches?
44. Is your tongue coating often thick?
45. Do you always have a bad appetite (don't want to eat)?
46. Do you often snack?
47. Do you always gobble up when you eat?
48. Do you often have an upset stomach or do you sometimes have nausea and vomiting?
49. Do you often feel vomiting (bloated abdomen) after meals?
50. Do you often burp after meals or do you have heartburn and sour water?
51. Do you often suffer from stomach problems?
52. Do you have indigestion?
53. Does severe stomach pain make you often have to bend over?
54. Do you feel persistent stomach discomfort?
55. Does anyone in your family have stomach problems?
56. Has the doctor said that you have gastric or duodenal ulcer disease or do you often feel stomach pain after meals or on an empty stomach?
57. Do you often have diarrhea (diarrhea)?
58. Did you have severe bloody stools (black stools or visible blood in the stool) when you had diarrhea or did you have mucus (sticky substance) in your stools when you had diarrhea?
59. Have you ever been troubled by intestinal parasites?

60. Do you often have severe constipation (dry stools)?
61. Do you have hemorrhoids (anal pain, discomfort during bowel movements, or
blood on the stool surface)
or dripping blood after defecation)?
62. Have you ever suffered from jaundice (yellowing of eyes, skin, urine)?
63. Have you ever had severe liver and gall
disease?

E

64. Do you often have joint swelling and pain?
65. Do your muscles and joints often feel stiff or stiff?
66. Do you often experience severe pain in your arms or legs?
67. Does severe rheumatism make you incapacitated or do you have tight shoulders
and neck muscles?
68. Does anyone in your family suffer from rheumatism?
69. Are your feet weak and sore making your life seriously inconvenient or do you often feel sore feet and legs?
70. Is your low back pain to the point where you cannot continue to work?
71. Are you troubled by serious functional loss or deformity (abnormal morphology) of your body?

F

72. Is your skin very sensitive to temperature, pain or tenderness or do you often have small subcutaneous hemorrhages (small red spots)?
73. Do incisions in your skin usually heal (grow well) easily?
74. Do you often blush?
75. Do you sweat profusely even in cold weather?
76. Does severe itching (itching) bother you?
77. Do you have frequent rashes (wind bumps or rashes) or do you sometimes have a puffy face?
78. Are you often troubled by boils
(pustules)?

G

79. Do you often suffer from severe headaches?
80. Do you often feel pain in life due to headaches and heavy hair?
81. Is headache common among your family members?
82. Do you have a fever and a cold?
83. Do you often have bouts of severe dizziness?
84. Do you often faint?
85. Have you fainted more than twice?
86. Do you have frequent numbness or tremors in any part of your body?

87. Have you ever been paralyzed (complete or partial loss of sensation and movement) in any part of your body?
88. Have you ever lost consciousness (don't know anything) after being knocked out?
89. Do you sometimes feel twitching (sudden and rapid muscle twitching) in your
head, face, and shoulders
Sleep?
90. Have you ever had a craziness (epileptic seizure, also called shofar craze)?
91. Are there any epilepsy patients in your family?
92. Do you have a severe habit of biting your nails?
93. Are you troubled by stuttering or stuttering or have you even had difficulty speaking due to an inflexible tongue?
94. Do you suffer from nocturnal wandering (walking around during sleep and not being able to recall things you did while asleep afterwards)?
95. Have you wet the bed lately?
96. Did you wet the bed at the age of 8- 14 (primary and secondary)?

H

(male answer)
97. Have you ever had some serious problem with your genitals
98. Do you often have genital pain or tenderness (pain at the touch)?
99. Have you ever received genital treatment?
100. Has the doctor said you had prolapse (prolapse of the rectum outside the anus)?
101. Have you ever had hematuria (painless)?
102. Have you ever been troubled by difficulty urinating?

(Female answer)
97. Do you often have dysmenorrhea (stomach pain during and before and after menstruation)?
98. Do you often get sick or feel weak during your menstrual period?
99. Do you often have periods of bed rest or do you have vaginal bleeding in addition to menstrual periods?
100. Do you often have persistent severe flushing and sweating?
101. Do you often feel anxious during your menstrual period?
102. Are you often troubled by abnormal leucorrhea (white vaginal mucus)? (answered by both men and women)
103. Do you get up every night to urinate?
104. Do you often urinate frequently during the day?
105. Do you often have a burning sensation (burning pain) when you urinate?
106. Do you sometimes have urinary incontinence (inability to consciously control urination)?

107. Has the doctor said that your kidneys and bladder are sick?
108. Do you often feel very tired?
109. Does work exhaust you?
110. Do you often wake up in the morning feeling tired and exhausted?
111. Do you feel tired after a little work?
112. Are you often tired and unable to eat?
113. Do you have severe neurasthenia?
114. Does anyone in your family suffer from neurasthenia?

J

115. Do you often get sick?
116. Are you often bedridden due to illness?
117. Are you always in poor health?
118. Do others think you are infirm?
119. Is there anyone in your family who is susceptible to illness?
120. Have you ever been unable to work due to severe pain?
121. Are you always overwhelmed by worrying about your health?
122. Are you always sick and unhappy?
123. Do you often feel unfortunate because of poor health?

K

124. Have you ever had scarlet fever?
125. Have you ever had rheumatic fever or pain in your limbs as a child?
126. Have you ever suffered from malaria?
127. Have you been treated for severe anemia?
128. Have you received STD treatment?
129. Do you have diabetes?
130. Has the doctor ever said you have a goiter (thick neck disease)?
131. Have you received treatment for tumor or cancer?
132. Do you have any chronic disease or have you ever been exposed to atomic radiation?
133. Are you thin (weight loss)?
134. Are you obese (gain weight)?
135. Has any doctor said that you have varicose veins in the legs (exposed superficial tendons of the legs)?
136. Have you been hospitalized for surgery?
137. Have you ever had a serious trauma?
138. Do you often have minor accidents and injuries?

L

139. Do you have difficulty falling asleep or not being able to wake up easily or do you often dream?

140. Are you unable to relax (rest) on a regular basis every day?
141. Is it difficult for you to exercise regularly every day?
142. Do you smoke more than 20 cigarettes a day?
143. Do you drink white sprinkles more than twice a day?
144. Do you drink tea and coffee more than the average person?

M

145. Do you sweat a lot or tremble a lot when you take a test or when you are questioned?
146. Are you nervous and trembling when approaching your supervisor?
147. Are you overwhelmed when your superiors watch you work?
148. Do you ever get completely confused when you have to do things quickly?
149. Do you have to be slow to avoid mistakes?
150. Do you often misunderstand (understand) the instructions or intentions of your superiors?
151. Are unfamiliar people or places intimidating you?
152. Do you panic because of loneliness when you have no acquaintances around?
153. Do you always have trouble making up your mind (hesitating)?
154. Do you always want someone by your side to give you advice?
155. Do others think you are a very stupid person?
156. Are you bothered to eat anywhere other than your own home?

N

157. Do you also feel lonely and sad at parties?
158. Do you often feel unhappy and depressed (low mood)?
159. Do you cry often?
160. Do you always feel miserable and depressed (disappointed)?
161. Do you feel completely hopeless about life?
162. Do you often want to die (a death thing)?

O

163. Are you often troubled (scouring)?
164. Is there anyone in your family who is frowning?
165. Does any little thing make you nervous and tired?
166. Do people think you are a neurotic (nervous, excitable) person?
167. Are there any neurotic people in your family?
168. Have you ever suffered from neurasthenia?
169. Has anyone in your family ever had a nervous breakdown?
170. Have you seen a doctor in a mental hospital (because of your mental problems)?
171. Has anyone in your family visited a mental hospital (because of their mental problems)?

P

172. Are you often shy and nervous?
173. Are there any shy and nervous people in your family?
174. Are your feelings vulnerable?
175. Are you always upset when you are criticized?
176. Do people think you are a picky person?
177. Are you often misunderstood?

Q

178. Do you have to be wary even with your friends (do not let your guard down)?
179. Do you always do things on a whim?
180. Are you easily annoyed and irritated?
181. Will you collapse if you don’t keep restraining yourself?
182. Does a little unhappiness make you nervous and lose your temper?
183. Do you get angry easily when others direct you?
184. Do others often displease and irritate you?
185. Get angry when you can't get what you need right away?
186. Do you often lose your temper?

R

187. Do you often tremble and tremble?
188. Are you often nervous and anxious?
189. Are you startled (jumping up or shaking violently) by sudden sounds?
190. Do you tremble and go weak with fright whenever someone calls out to you?
191. Are you afraid (fear) of sudden movements at night?
192. Do you often wake up from nightmares?
193. Do you have some kind of terrifying (horrible) thought that recurs in your head?
194. Do you often feel suddenly intimidated (fear) for no reason?
195. Do you often have sudden cold sweats?
